# Change in Advertising Exposure of Unhealthy Commodities After the Implementation of an Outdoor Advertising Restrictions Policy in Bristol, UK: A Descriptive Study

**DOI:** 10.1007/s11524-026-01095-x

**Published:** 2026-06-24

**Authors:** Toumpakari Z., Buckland G., Eyles E., Sillero-Rejon C., Jago R., Cummins S., Nobles J., Harding S., Horwood J., Nairn A., Hollingworth W., Blake S., Anderson J., Paul C., Whitlow H., Cejas Hidalgo O., Stone G., de Vocht F.

**Affiliations:** 1 Centre for Exercise, Nutrition and Health Sciences, School for Policy Studies, University of Bristol, Bristol, UK; 2 Bristol Medical School, Population Health Sciences, University of Bristol, Bristol, UK; 3 NIHR Applied Research Collaboration West (NIHR ARC West), Bristol, UK; 4 Population Health Innovation Lab, Department of Public Health, Environments & Society, London School of Hygiene & Tropical Medicine, London, UK; 5 School of Health, Leeds Beckett University, Leeds, UK; 6 Bristol Hub for Gambling Harms Research, University of Bristol, Bristol, UK; 7 Centre for Research in Health and Social Care, School of Policy Studies, University of Bristol, Bristol, UK

**Keywords:** Advertising, Exposure, HFSS, Unhealthy commodities, Public Health, Policy Evaluation, Commercial determinants, CDOH, Nutrition

## Abstract

In 2021, Bristol, UK, implemented a city-wide policy restricting advertising of unhealthy commodities on council-owned sites. We aimed to describe advertising exposure before and after the implementation of the policy in Bristol. We collected data from bus shelters in Bristol (N = 283) and neighboring South Gloucestershire (SG, comparator, N = 65), using in-person and Google Street View audits, before (T0, 02/2022 – 07/2022) and after (T1, 04/2023 – 07/2023 and T2, 11/2023 – 02/2024) policy implementation. We captured unhealthy commodity adverts on display; high in fat, sugar or salt (HFSS) products (defined using the UK Nutrient Profiling Model), alcohol, gambling or payday loans; information on brands; whether they appealed to children or adolescents and split analyses by area deprivation and proximity to schools. At T0, unhealthy commodity advertising in both areas was relatively low, however the policy successfully reduced unhealthy commodity advertising in Bristol at T2 (11.3% to 0.8%), compared to an increase in SG (0.9% to 18.1%). Most restricted adverts in both areas displayed HFSS products (ranging 60–100%), especially fast food, with limited alcohol and gambling and no payday loan advertising observed. A few adverts displaying non-compliant products were maintained in Bristol but advertisers also switched to advertising alternative, compliant products. Minimal unhealthy commodity advertising appealed to children/adolescents (1.1–8.8%) or appeared within 100m of schools (N = 2 adverts), and no clear association with area deprivation was found. Findings suggest the need for formal monitoring processes to ensure policy adherence and assess unhealthy commodity advertising in the entire outdoor space to inform potential expansion of such policies.

**Supplementary Information:**

The online version contains supplementary material available at https://doi.org/10.1007/s11524-026-01095-x.

## Introduction

The creation of health promoting environments is a global priority for addressing rising non-communicable diseases [1]. A key aspect of the current environment is the advertising and marketing of alcohol, gambling and foods and drinks high in fat, sugar or salt (HFSS), which impacts attitudes and consumption/use of these commodities [2–5]. Availability of payday loans, i.e., display of a company offering short-term, high-interest loans, has also been associated with psychological health, obesity and diabetes [6, 7]. Governments increasingly implement responsible marketing policies, including advertising restrictions [8, 9] to help reduce exposure to these products [10–12].

Advertising restriction policies often focus on restricting child-targeted advertising of HFSS foods/drinks on traditional broadcast media, such as television [12, 13]. However, children and adults are exposed to advertising of unhealthy commodities across multiple settings, including outdoor public spaces, where the content of advertising remains comparatively unregulated, in the UK [10].

Outdoor advertising can be defined as ‘commercially available and physically rentable assets situated outside the home in both public and commercial places [14] and includes billboards, hoardings, bus shelters, and transport facilities, across public and private spaces. It is a major advertising sector due to its extensive reach and high visibility [15]. HFSS products are commonly marketed on outdoor advertisements; a scoping review reported that 22% of outdoor adverts were food related, of which 83% were unhealthy [3]. In the UK, HFSS adverts accounted for 33% of bus shelter adverts in Scotland [16] and 16% of bus shelter adverts in a deprived area of Northern England [17].

Cross-sectional evidence correlates HFSS outdoor advertising with increased sugary drink purchases in adults [18], fast-food purchases in adolescents [19] and consumption of certain HFSS products in pre-school children [20]. Systematic reviews also show that exposure to gambling and alcohol advertising was associated with increased intention and behavior to gamble [4]_,_ and current or higher-risk drinking in adolescents [5]. Therefore, advertising restriction policies may reduce consumption of HFSS products and other unhealthy commodities, such as gambling and alcohol.

Governments across the world have implemented policies to restrict unhealthy food and drink advertising in outdoor spaces, however, very few formal evaluations have been conducted to understand their impact on consumption behavior or health [21]. In 2022, in England, only a third of councils, i.e., local Government organisations (hereby councils), (N = 106) had an advertising and sponsorship policy (mostly applying to all outdoor advertising space owned by the councils), predominantly targeting gambling (N = 84), alcohol (N = 78) and less so HFSS products (N = 25) [9]. However, variability in policies’ definitions and application result in lack of consistent specification and implementation.

In 2019, a new outdoor advertising policy was implemented across the Transport for London (TfL) estate, restricting advertising of HFSS products, and which was associated with an average relative reduction in purchased energy of 1,000 cal less per household per week [22]. However, the evaluation did not objectively assess advertising exposure before and after the implementation of the policy. Understanding change in advertising exposure is a crucial element of the causal mechanism, as any behavioral impact of the policy is likely to be linked to the change in the advertising environment.

In November 2021, Bristol introduced a new Advertising and Sponsorship Policy (hereby ‘policy’), restricting advertising of HFSS products, as well as alcohol, gambling and payday loans on outdoor council-owned spaces [23]. The policy was fully implemented by the summer of 2022, due to existing advertising contracts finishing at different times in the year. The Bristol Evaluation of Advertising Restrictions (BEAR) study was developed to assess implementation policy and impact [24, 25]. As part of the evaluation, we aimed to describe change in unhealthy commodity advertising exposure in Bristol and neighboring comparator area of South Gloucestershire (SG) before and after policy implementation and explore unhealthy commodity advertisement density by area deprivation and proximity to schools.

## Materials and methods

The protocol for this study has been previously published (https://osf.io/7yc9s/). Briefly, we collected data in Bristol, UK and, as a comparator, the neighboring area of SG at three timepoints and different seasons; before the implementation of the policy in Bristol ‘T0’ (02/2022–07/2022), one year after the implementation of the policy ‘T1’ (04/2023–07/2023) and 6 months later ‘T2’ (11/2023–02/2024). Both councils provided a list of all council owned outdoor advertising sites which included bus shelters, alongside latitude and longitude co-ordinates, encompassing 283 locations in Bristol and 65 in SG. The difference in number of locations is mostly due to differences in advertisement and bus shelter density but also results from differences in how much of the advertising space is owned by each council. A map of audited bus shelters, as well as small-area (lower-super output area (LSOA)) [26] deprivation measures (according to the Index of Multiple Deprivation (IMD) [27]), in both councils is presented in **Supplementary Fig. 1**.

Adverts on display were captured using both in-person auditing (T0, T1 and T2) and Google Street View (GSV) (T0 and T1), to improve data collection accuracy due to geocoding errors, obstructed sites and safety concerns, using a previously established approach [28]. For in-person auditing, fieldworkers (T0: N = 3, T1: N = 3, T2: N = 2) traveled to each advertising site and photographed all advertisement panels on display using phones. Similarly, using GSV, fieldworkers took screenshots of all bus shelter adverts on display. In the event of multiple or rolling advertising panels, each advert was captured as an individual image.

## Coding of Advertisements

Coding of adverts was based on the INFORMAS outdoor advertising protocol [29] and the WHO TV monitoring protocol [30]. Coding was conducted by the fieldworkers who collected data at each timepoint. Based on the Bristol Advertising and Sponsorship policy [31], adverts for unhealthy commodities were those which displayed any HFSS product, any alcoholic drinks, any form of gambling (except for the National Lottery or for small or large society or council lotteries) or payday loans. All adverts were coded according to four dimensions: 1) advertising unhealthy commodities (yes/no); 2) product type (food, non-alcoholic drink, alcoholic drink, gambling, pay-day loan); 3) brand advertised; and 4) displaying products appealing to children or adolescents < 18 years old (yes/no).

Adverts that displayed foods or non-alcoholic drinks, were categorised into 12 food/drink groups (Supplementary Table 1). Some of these food/drink groups were the same as in the evaluation of TfL advertising restrictions, for comparability purposes, whereas additional food groups were also used matching the ones in the UK National Diet and Nutrition Survey [31] ^(32)^. Using ‘researcher judgment’[30], we captured adverts that displayed products appealing to children or adolescents, i.e., products specifically targeted to children/adolescents, such as kids’ yoghurt, as well as adverts that would display images/characters appealing to young people, cartoon characters, young people, etc. We also captured the creative strategies used in these adverts [30], e.g., use of celebrity endorsers, musical jingles, brand equity characters, etc. More details on the coding process are presented in Supplementary File.

## Nutrient Profiling of Foods/Drinks Advertised

Adverts that displayed foods and non-alcoholic drinks were classified as HFSS or not, based on the UK Nutrient Profiling Model (NPM) [32] 2004–2005. Information on energy (kJ), saturated fat (g), sugar (g), sodium (mg), fiber (g), protein (g) and fruit, vegetables and nuts (%) per 100 g of product was obtained primarily from product manufacturers’ websites, major UK retailers or the McCance and Widdowson’s Composition of Food Integrated Dataset (CoFID) [33]. For products with nutritional information presented per portion/serving, these were converted to grams. For some food/drink products, it was not possible to identify information on their serving size/grams, therefore the NPM score was marked as missing. Food products that received a score ≥ 4 and non-alcoholic drinks that received a score ≥ 1 were classified as HFSS.

## Deprivation and Proximity to Schools

The level of advertising sites’ relative deprivation was captured IMD 2019 [27]. LSOAs were classified into deciles by IMD, which was used to calculate number of unhealthy commodity adverts in each decile. We assessed proximity of adverts to primary and secondary schools in Bristol and SG, using three different buffer zones, i.e., within 100 m according to the Advertising Standards Authority [34], within 200 m because some companies have a voluntary policy to not advertise within this distance to schools [35] and within 400 m, due to London Mayor’s prior commitment to restrict advertising within 400 m near schools [35]. The distance thresholds are cumulative, meaning that the 100 m, 200 m and 400 m bounds encompass the areas within their shorter bounds (e.g., 100 m would be within 0-100m of a school). A list of all primary and secondary schools (both state and private) was obtained from the UK government’s website [36].

## Analysis

Analysis was conducted using Stata (version 18.5 for Windows) and R (version 4.4.2 for Windows). Descriptive statistics are presented as raw frequencies and percentages. To investigate potential displacement of adverts from Bristol to SG, we compared advertising in bus shelters that were close to Bristol/SG borders (within 400 m) and all remaining bus shelters. The primary aim was to describe change in unhealthy commodity advertising exposure, hence we did not conduct statistical testing (also due to insufficient sample sizes of unhealthy commodity adverts at T1 and T2 (relevant sample size < 25)). The geographic data were collected from primarily Open Government License sources, including IMD 2019 [27] and the location of the schools [36]. The computer code used to generate the buffers and determine whether an advertisement was within a buffer distance of 100/200/400m can be found on GitHub https://github.com/eyles-ec/useful-code-bits/tree/main/Geographic%20data%20preparation/Schools%20and%20advertisement.

For the main analysis, we used in-person data, due to better image quality, which allowed more adverts to be coded. Preliminary coding of GSV-collected adverts showed poor image quality limiting its use to only T0 and T1. Additionally, nearly half of the GSV images collected at T1 (N = 523, 47%) were showing adverts before the implementation of the policy (dated 2018–2021), which prevented a before-and-after comparison of advertising exposure. However, some overall descriptives using GSV data are also reported for comparison.

## Results

Using in-person audit, we identified 859 advert panels in Bristol and 114 in SG at T0, 1295 in Bristol and 128 in SG at T1 and 1636 in Bristol and 160 in SG at T2 (Supplementary Table 1). The difference in the number of advert panels is due to an increase in the number of panels in certain bus shelters, as well as more rotating advert panels, which were treated as individual adverts. Descriptives using GSV audit are presented in Supplementary Table 2. A similar number of adverts was identified at T0 (Bristol: N = 876, SG: N = 44) and T1 (Bristol: N = 986, SG: N = 124), compared to the in-person audit.

**Fig. 1:**
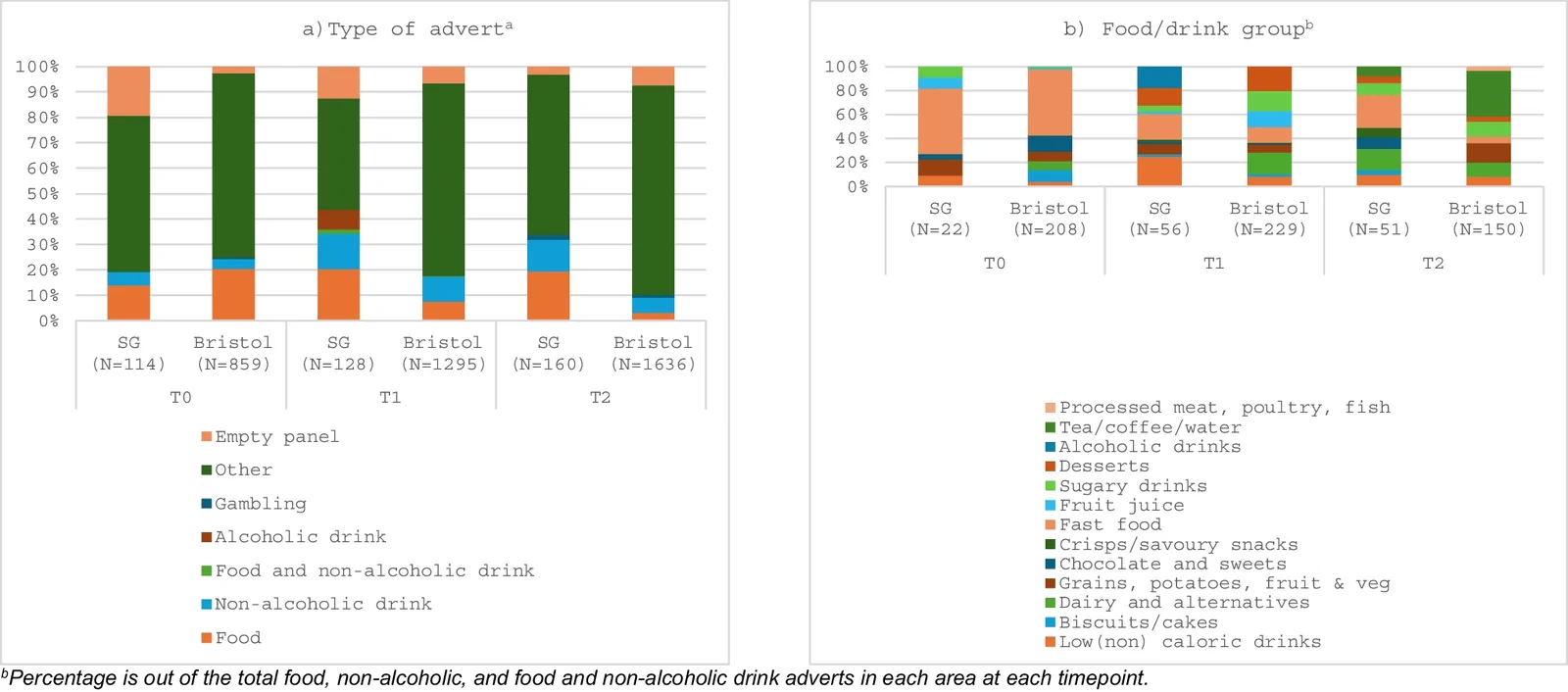
Description of **a**) type of adverts and **b**) advertised food/drink categories, in overall adverts, in Bristol and SG, across T0, T1 and T2. ^b^Percentage is out of the total food, non-alcoholic, and food and non-alcoholic drink adverts in each area at each timepoint. ^a^Percentage is out of total adverts in each area at each timepoint

Figure 1 and Supplementary Table 1 present a description of the overall outdoor advertising landscape in Bristol and SG for HFSS and non-HFSS products combined. In both areas, across the 3 timepoints, most adverts displayed non-food/drink products ranging from 61.4–82.4% of total adverts (Fig. 1a). At T0, in both Bristol and SG, food adverts were the second highest (20.5% and 14% respectively), mostly displaying fast food products (55.3% and 54.6% respectively). At T1, in Bristol, there was a large decrease in overall food adverts (−12.9%), mainly driven by a drop in fast-food adverts (−42%), whereas more adverts displayed non-alcoholic drinks (+ 6.2%), especially sugary drinks (+ 15.4%). In SG, unlike Bristol, number of food adverts increased (+ 6.3%). At T2, in Bristol, food adverts continued to decrease (−4.4%) and there was also a decrease in non-alcoholic drink adverts (−4%). The few gambling adverts that were identified (1.1%) in Bristol at T2 were exempt from restrictions, e.g., advertising the National Lottery. In SG, the number of food and non-alcoholic drink adverts at T2 remained similar to T1 (19.4% and 12.5% respectively) and as in Bristol, few gambling adverts, advertising the National Lottery, were identified (1.9%).

**Fig. 2:**
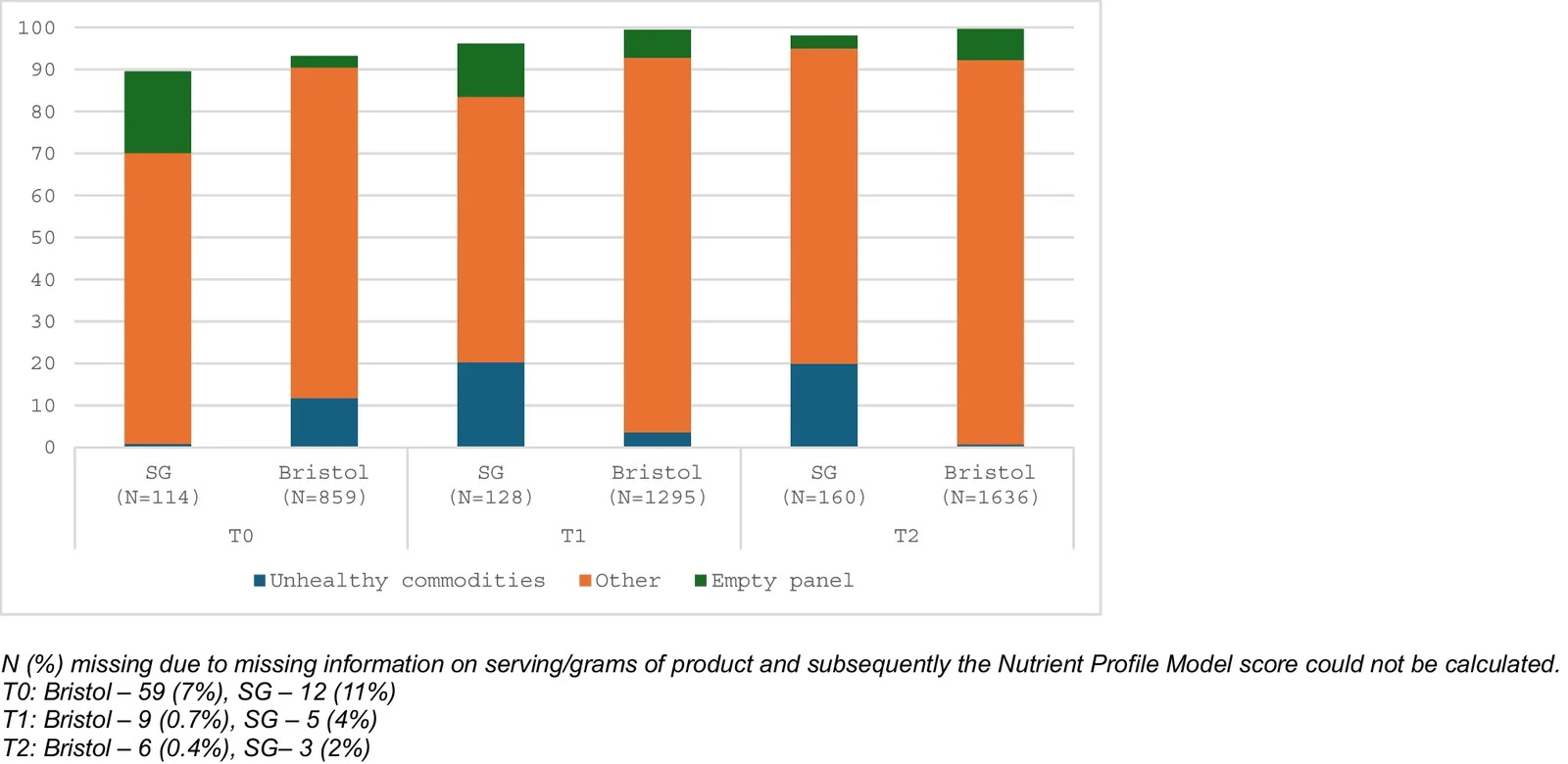
Percentage of adverts for unhealthy commodities and other products in Bristol and South Gloucestershire, at T0, T1 and T2. N (%) missing due to missing information on serving/grams of product and subsequently the Nutrient Profile Model score could not be calculated. T0: Bristol – 59 (7%), SG – 12 (11%). T1: Bristol – 9 (0.7%), SG – 5 (4%). T2: Bristol – 6 (0.4%), SG– 3 (2%)

Figure 2 shows the proportion of adverts for unhealthy commodities (including HFSS products) in Bristol and SG (out of the total number of adverts in each council) across the three timepoints. At T0, in Bristol, 11.3% of the adverts displayed unhealthy commodities, most of which advertised fast food (46.9%) (Supplementary Table 3). In SG, at T0, only one advert for unhealthy commodities was identified (0.9%), displaying chocolate and sweets. At T1, adverts for unhealthy commodities in Bristol dropped to 3.6%, although HFSS sugary drink adverts increased (+ 62%) (Supplementary Table 3). In contrast, in SG, adverts for unhealthy commodities at T1 increased to 20.3% displaying mostly alcoholic drinks (36%). At T2, in Bristol, adverts for unhealthy commodities dropped even further to 0.8%, whereas in SG, the number of adverts for unhealthy commodities remained similar to T1 (18.1%), although displaying more fast food (27.6%). No adverts of pay day loans were identified in either area across the three timepoints. Gambling adverts were identified at T0 and T1 (Fig. 1a**)**, however, only one was considered unhealthy commodity in Bristol at T0. Alcohol adverts were only present in SG at T1 (N = 10) (Fig. 1a).

## Advertised Brands

The top five most commonly advertised brands in Bristol and SG are shown in Supplementary Table 4. For adverts not displaying unhealthy commodities, common organisations/brands advertised, included charities (e.g., Macmillan Cancer Support), Transport/Travel (e.g., TravelWest, Metrobus), and food/drink brands that would advertise policy compliant, non-HFSS products (e.g., Muller yoghurt, Cawston Press juice, Diet Coke).

At T0, in Bristol, almost half of the adverts for unhealthy commodities displayed fast-food chains (44.6%), i.e., McDonald’s, Burger King and KFC and food/drink products (44.6%), such as LU and Cadbury, whereas in SG only Tesco advertised HFSS products. At T1 and T2, in Bristol, some HFSS fast-food products by McDonald’s and Subway were still being advertised (T1: N = 7, T2: T = 2), as well as HFSS products by Coca Cola, San Pellegrino, Oxo and Muller (T1: N = 33, T2: N = 8). In addition, McDonald’s and Coca Cola also switched to advertising non-HFSS products, e.g., low(non)-caloric drinks, whereas brands like LU or Cadbury stopped advertising entirely. During the same timepoints, in SG, adverts of HFSS fast-food products by McDonald’s and Subway appeared (T1: N = 5, T2: N = 11), as well as HFSS food/drink products by Coca Cola, Pepsi and Nestle (T1: N = 11, T2: N = 10). Alcoholic drinks were advertised by Strongbow and Thatcher’s in SG at T1 (N = 10). Only few bus shelters were identified close to the Bristol-SG border (Bristol: N = 25, SG: N = 11), which included few adverts for unhealthy commodities (up to N = 5) across the three timepoints, (**Supplementary Table 5**), suggesting no displacement near the borders.

## Adverts Appealing to Children or Adolescents

**Fig. 3:**
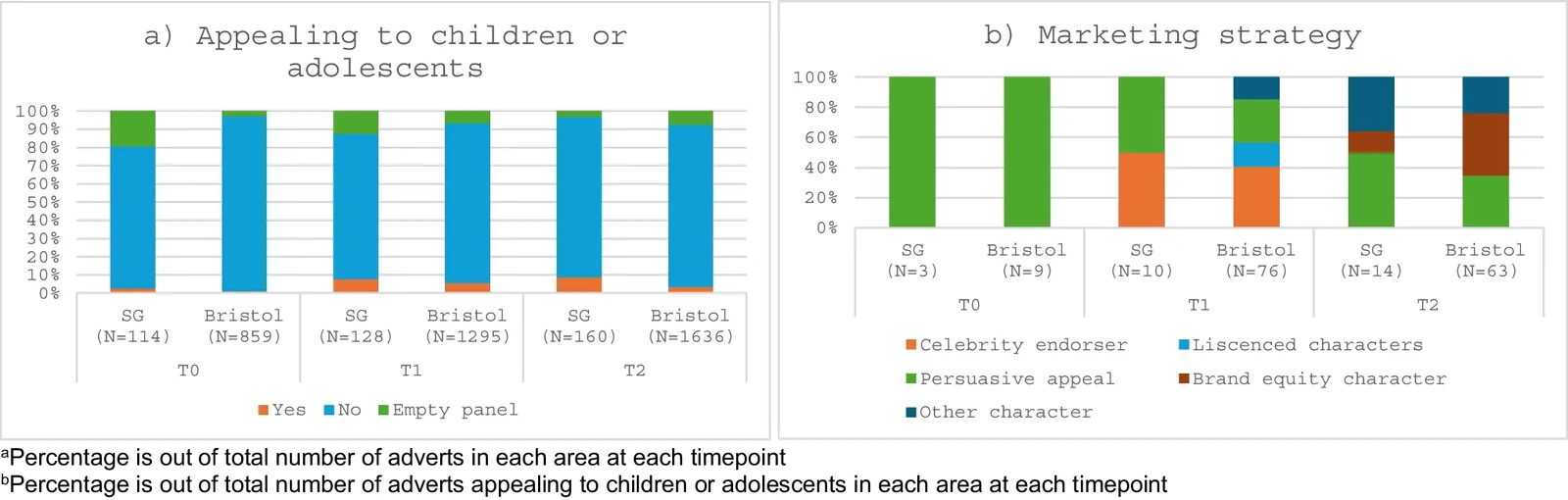
Adverts **a**) appealing to children or adolescents and **b**) marketing strategies used in these adverts, in Bristol and SG at T0, T1 and T2. ^a^Percentage is out of total number of adverts in each area at each timepoint. ^b^Percentage is out of total number of adverts appealing to children or adolescents in each area at each timepoint

At T0, few adverts were appealing to children or adolescents (Bristol: 1.1%, SG: 2.6%) (Fig. 3). This increased to 5.6% and 7.8% in Bristol and SG, respectively at T1. At T2, there was a decrease in Bristol (3.4%), whereas there was a slight increase in SG (8.8%). Use of persuasive appeal, celebrity endorsers and brand equity characters were amongst the most common marketing strategies used in these adverts **(**Fig. 3). However, in both areas, across all timepoints, only very few adverts for unhealthy commodities were appealing to children or adolescents (up to 10 adverts) (Supplementary Fig. 2).

## Advertising Density and Proximity to Schools

**Fig. 4:**
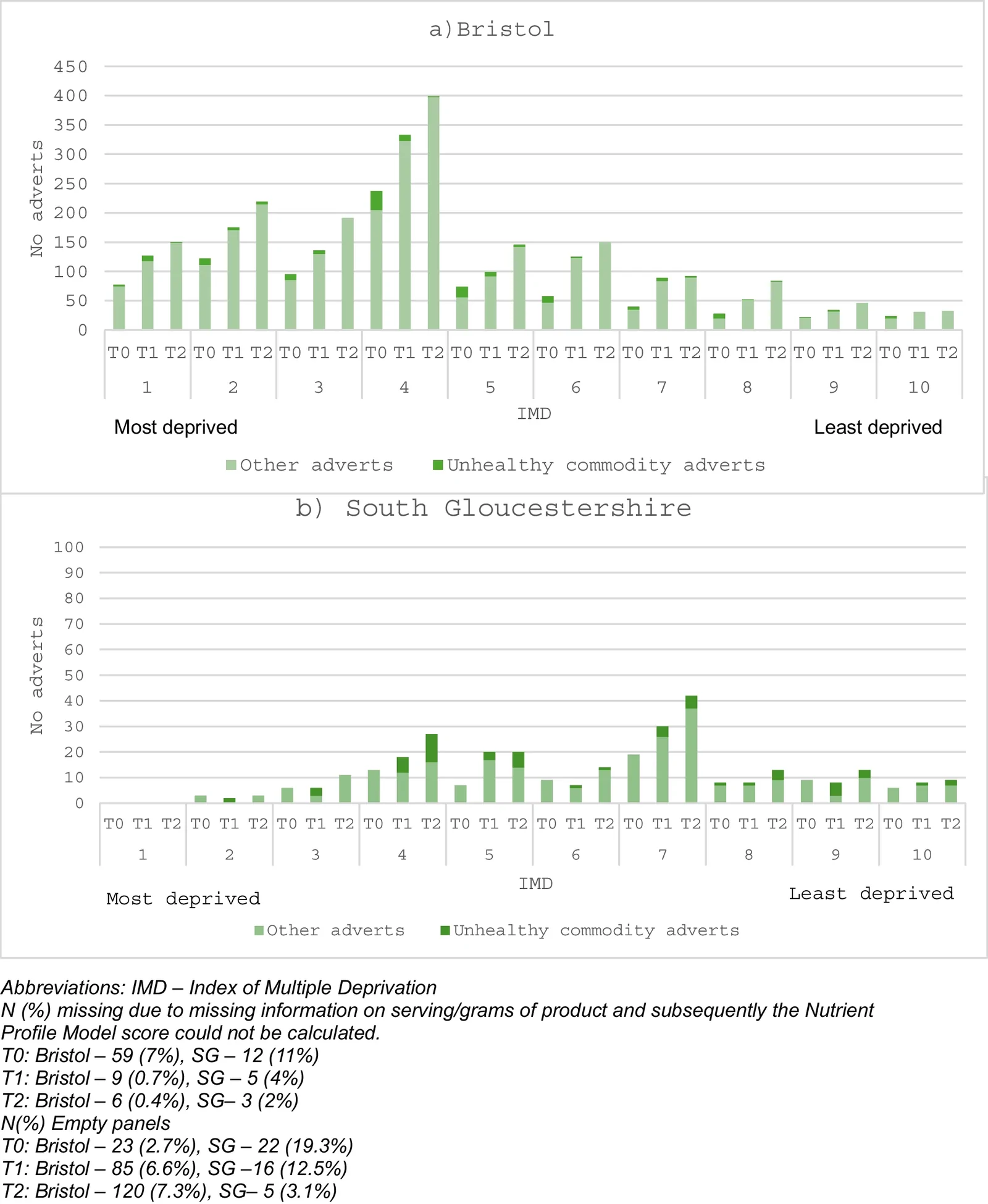
Number of adverts for unhealthy commodities and other products in **a**) Bristol and **b**) South Gloucestershire (SG) by deprivation (IMD) decile, at T0, T1 and T2. Abbreviations: IMD – Index of Multiple Deprivation. N (%) missing due to missing information on serving/grams of product and subsequently the Nutrient Profile Model score could not be calculated. T0: Bristol – 59 (7%), SG – 12 (11%). T1: Bristol – 9 (0.7%), SG – 5 (4%). T2: Bristol – 6 (0.4%), SG– 3 (2%).N(%) Empty panels. T0: Bristol – 23 (2.7%), SG – 22 (19.3%). T1: Bristol – 85 (6.6%), SG –16 (12.5%). T2: Bristol – 120 (7.3%), SG– 5 (3.1%)

There was no clear pattern in the advertising of unhealthy commodities in Bristol or SG according to deprivation level **(**Fig. 4). In Bristol, at T0, more adverts in total (28%) and for unhealthy commodities (33%) were found in middle deprived areas, i.e., IMD = 4. At T1 and T2, in Bristol, there were more adverts for unhealthy commodities in middle and most deprived areas compared to least deprived areas. In contrast, at T1 and T2, in SG, there were more adverts for unhealthy commodities in middle and least deprived areas compared to most deprived area.

Supplementary Table 6 presents total and unhealthy commodity advertising near schools in Bristol and SG, across the three timepoints. In both areas, few adverts were found within 100 m of a school, and only two displayed HFSS products (SG: N = 1 at T1, Bristol: N = 2 at T2). Within 200 m of any school, no adverts for unhealthy commodities were found at T0 in either area, but a few HFSS adverts were identified in both Bristol and SG at T1 (up to N = 6). Around half of the total adverts in Bristol and SG were within 400 m of a school across the three timepoints. In Bristol, at T1 and T2, unhealthy commodity adverts were identified within 400 m of a school (T1: N = 24, T2: N = 8). In SG, unhealthy commodity adverts within 400 m of a school increased from N = 1 to N = 13 at T2, including three adverts of alcohol.

## Discussion

We objectively assessed and described outdoor unhealthy commodity advertising before and after the implementation of advertising restrictions in Bristol and comparator area of SG. At T0, unhealthy commodity advertising in Bristol and SG council owned bus shelters was low. The policy successfully reduced unhealthy commodity advertising in Bristol, whereas exposure increased in SG. Most unhealthy commodity adverts across all timepoints displayed HFSS products, most commonly fast-food, sugary drinks and chocolate and sweets. Adverts continued to display some unhealthy commodities in Bristol at T1 and T2, mainly sugary drinks and fast food, although these were reduced at T2. Brands with HFSS product lines also switched to healthier alternatives, such as low-(non) caloric drinks. There was minimal alcohol and gambling advertising and no advertising of pay-day loans. Very few unhealthy commodity adverts were appealing to children or adolescents, and few were near schools (100m). There was no clear pattern between outdoor advertising and level of deprivation, with most unhealthy commodity adverts located in middle deprived areas.

Our findings are consistent with previous research in the North of England, also showing low unhealthy commodity advertising in the outdoor space (16%) [28] and in Auckland, New Zealand, where 13% of bus shelter adverts displayed non-core products, i.e., foods/drinks that are surplus to requirements [37]. Other studies in the North of England and Scotland, however, have shown higher number of outdoor adverts of unhealthy commodities (mostly HFSS products), ranging from 30–35% [16, 38, 39]. Differences could be attributed to sampling of different councils, variability in outdoor advertising definition, e.g., all outdoor adverts vs. council owned space, as well as differences in the way compliant food/drink adverts were defined. Bristol’s low pre-policy unhealthy commodity advertising may reflect its previous initiatives to create a healthy food environment, for example, it adopted the Healthy Weight Declaration in 2020, (which includes actions on restricting unhealthy food marketing) [40] and was awarded Gold Sustainable Food city in 2021. In addition, we only assessed council owned sites, without capturing other forms of outdoor advertising, such as advertising in retail (e.g., digital billboards in shopping centers) and transport (e.g., on buses, rail stations, and airports) [3], which resulted in underestimating the total exposure to outdoor advertising. Future research should aim to assess all forms of outdoor advertising to understand its true prevalence and associations with behavior and health. Finally, not all previous studies have used objective criteria, e.g., the UK NPM, to determine food/drink adverts’ suitability to be advertised [16, 38, 39]. They have instead used broad food groups, such as fast food, frozen desserts, without considering the nutritional quality of each advertised product, resulting in a potential misestimation of HFSS advertising.

Fast-food chains were the most advertised unhealthy commodities in Bristol at T0, similarly to previous research in the UK and elsewhere [3, 28]. This is not surprising given that fast-food chain McDonald’s was the largest outdoor advertiser in 2024, having spent £86.3m [41]. Although fast-food advertising decreased in Bristol after the policy came into place, there were still a few non-compliant fast-food and other food/drink adverts present (e.g., from McDonalds, Subway, Coca Cola and San Pellegrino). A lack of formal monitoring process could account for some remaining non-compliant adverts, as well as challenges for the council to keep on track with long-term advertising contracts [41, 42]. Councils could benefit from introducing formal processes to monitor adherence to the policy, such as having designated teams within the council being responsible for policy adherence [43], rather than relying on ad-hoc processes of complaints being raised for non-compliance [42]. Financial consequences for non-compliance have also been suggested by those involved in the development of the Bristol policy [42] and have been enforced in advertising policies elsewhere [44]. In addition, small and medium-sized businesses may not have as much knowledge and capacity to spend resource on adjusting to the policy [43], it is therefore imperative that local councils help these businesses understand policy requirements and how to meet them.

Our findings suggest that advertisers used certain tactics to respond to the policy. That there was a decrease in unhealthy commodity advertising in Bristol from T0 to T2 but an increase in the neighboring area of SG, indicates an industry move to advertise unhealthy commodity products elsewhere, although displacement was not evidenced explicitly near the Bristol/SG border. In addition, some large brands (e.g., McDonald’s and Coca Cola) with HFSS product lines switched to advertising of healthier, compliant products, for example low-(non) caloric drinks. Brand exposure is a powerful strategy that can impact on food preference, even when healthier products are advertised [45], hence this could partly explain why no difference in purchasing of HFSS has been observed in Bristol after the policy was implemented [46].

Consistent with some previous research we did not observe a clear advertising pattern in relation to level of deprivation in either area [3, 28, 38, 39]. Other studies, however, have shown higher exposure to unhealthy food advertising in more deprived areas [16, 37] and in ethnic minority communities [3, 47]. Our findings showed more total and unhealthy commodity adverts in middle deprived areas, and in the case of SG, slightly more in the least deprived compared to most deprived areas. This may be due to outdoor advertising clustering in central, high transport areas. Future research should attempt to further explore the association between deprivation, and other socioeconomic indicators, to better understand associations with outdoor unhealthy commodity advertising.

Few adverts were appealing to children or adolescents, and even fewer were displaying unhealthy commodities. Previous research in the North of England has found a higher number of bus shelter adverts being appealing to children and adolescents (71.9%), 38.7% of which were classified as HFSS [28]. This could be due to our study not classifying as appealing, any food image [28], unlike previous research, but only those displaying children’s products and those using images/characters that would appeal to children/adolescents. Previous research has shown high unhealthy commodity advertising in places where children and adolescent may spend more time playing or socialising, like parks and sport fields [48], suggesting that additional outdoor places may also benefit from advertising restrictions to create healthy environments for this age group.

There was minimal unhealthy commodity advertising in close proximity to schools (100m), which could be due to existing voluntary policies restricting HFSS advertising within 100 m of a school [49], although the two adverts identified indicate there is not complete compliance with this policy. More non-compliant adverts (including HFSS and alcohol) were identified when distance from schools increased, similarly to previous research in New Zealand [37]. Advertising restrictions could therefore be expanded to wider zones around schools, as young people travel and can therefore be exposed to unhealthy commodity marketing beyond their immediate home or school neighborhood [50].

### Strengths and Limitations

To our knowledge, this is the first study that described changes in outdoor advertising exposure of unhealthy commodities before and after the implementation of an advertising restrictions policy. We used objective auditing tools and criteria to define compliance of food/drinks products with the policy, i.e., the UK NPM. We used a comparator area and collected post-policy implementation data across two timepoints and across different seasons (winter/spring vs. autumn) to account for differences in product advertising.

Limitations should also be acknowledged. Although using GSV in health research offers advantages, such as saving time, being low-cost and easy to use [51], images were often low quality and outdated, making them unsuitable for assessing whether adverts displayed unhealthy commodities or for describing change in exposure. The policy was introduced in November 2021 and was fully implemented by summer 2022, due to existing advertising contracts finishing at different times in the year. Baseline unhealthy commodity adverts could have therefore been slightly underestimated by contracts that had already finished up to the point of data collection. We conducted T0 and T1 data collections at similar times in the year. However, T0, unlike T1, overlapped with celebration periods, such as St. Valentines Day and Easter, so chocolate and confectionery advertising could have been higher. Nevertheless, the proportion of food adverts that came from these products at T0 was fairly low, i.e., 23%. Appeal to children was based on researcher judgement [30], as done previously, and could therefore have led to misestimation of the proportion of adverts appealing to this age group. We employed objective criteria, i.e., UK NPM, to assess whether food and drink adverts displayed unhealthy commodities. However, the UK NPM is only one model and like all models has limitations [52], for example, it may permit more products to be advertised compared to other models [28]. The updated NPM 2018 [53], may address some of these challenges as it is slightly stricter on energy and saturated fat and aligns with current recommendations on free sugars and fiber, hence some of the products classified as non-HFSS by NPM 2004–2005 may be classified as HFSS with the NPM 2018. Future research should therefore examine the use of alternative models to assess nutritional quality and advertising suitability.

## Conclusion

The outdoor advertising restrictions policy was successful in reducing unhealthy commodity advertising in Bristol, although pre-policy levels were already low. The increase in unhealthy commodity advertising in SG suggested some displacement to a neighboring area. After the policy was implemented, advertising of some non-compliant products continued, although to a lesser extent, or advertisers switched to advertising of compliant products. Our findings highlight the need to establish formal monitoring processes to ensure policy adherence, as well as assessing unhealthy commodity advertising in the entirety of outdoor advertising space to consider extending the policy accordingly. Additionally, we advocate that policymakers should recognise tactics that advertisers may use to respond to such policies and consider effective countermeasures to maximise the policy’s impact. Future evaluations of advertising restriction policies should include objective measures of exposure to understand changes in the environment and their impact on behavior.

## Supplementary Information

Below is the link to the electronic supplementary material.Supplementary file1 (DOCX 201 kb)
