# Supplementary material for: Change in Advertising Exposure of Unhealthy Commodities After the Implementation of an Outdoor Advertising Restrictions Policy in Bristol, UK: A Descriptive Study
Source: J Urban Health. 2026 Jun 24;103(4):742–55. doi: 10.1007/s11524-026-01095-x (PMC13524900; doi:10.1007/s11524-026-01095-x)
Supplement: Supplementary file 1 [file 11524_2026_1095_MOESM1_ESM.docx]

**Supplementary file**

**Details on advert’ coding process fieldworkers’ training**

All fieldworkers participated in 2x1-hour online training sessions about data collection and the advert coding process. Each fieldworker initially coded a sample of 20 adverts, which were then discussed as a group to resolve any queries and address any initial coding discrepancies. At the end of the coding process 10% of adverts were independently coded by each of the fieldworkers at the corresponding timepoint. Estimated agreement between fieldworkers across the three timepoints was 92%, 91% and 93% respectively, suggesting a high level of agreement ^(33)^.

Advert panels that were empty at the time of data collection were recorded as ‘Empty panel’. In the case of multiple advertised products, the brand of the biggest product was captured where possible, or if not clear, the first product on the top left of the advert^(34)^ . When using the GSV method, adverts that were illegible due to poor visual quality, were recorded as ‘Not viewable panel’. GSV adverts recorded as ‘Not viewable panel’ were recoded to missing.

**Supplementary** Figure 1: Map of council owned bus shelters across Bristol and South Gloucestershire lower-super output areas (LSOAs)^30^ and by level of deprivation according to IMD.

Source: Office for National Statistics licensed under the Open Government Licence v.3.0

Contains OS data © Crown copyright and database right 2025

Blue dots represent bus stops in Bristol and red dots show bus stops in SG.

Different shades of brown areas represent differences in LSOAs’ level of deprivation according to the Index of Multiple Deprivation^31^.

Abbreviations: IMD – Index of Multiple Deprivation

**Supplementary Table 1:** Description of overall adverts in Bristol and South Gloucestershire (SG) at T0, T1 and T2.

|  | T0 (n=973) | | | | T1 (n=1,423) | | | | T2 (n=1,796) | | | |
| --- | --- | --- | --- | --- | --- | --- | --- | --- | --- | --- | --- | --- |
|  | **02/2022 - 07/2022** | | | | **04/2023 - 07/2023** | | | | **11/2023 - 02/2024** | | | |
|  | **SG** | | **Bristol** | | **SG** | | **Bristol** | | **SG** | | **Bristol** | |
|  | **N** | **%** | **N** | **%** | **N** | **%** | **N** | **%** | **N** | **%** | **N** | **%** |
| Number of Total Adverts | 114 | 100 | 859 | 100 | 128 | 100 | 1295 | 100 | 160 | 100 | 1,636 | 100 |
| *Type of Advert* | | | | | | | | | | | | |
| Food | 16 | 14 | 176 | 20.5 | 26 | 20.3 | 98 | 7.6 | 31 | 19.4 | 53 | 3.2 |
| Non-alcoholic drink | 6 | 5.3 | 32 | 3.7 | 18 | 14.1 | 128 | 9.9 | 20 | 12.5 | 97 | 5.9 |
| Food and non-alcoholic drink | 0 | 0 | 0 | 0 | 2 | 1.6 | 3 | 0.2 | 0 | 0 | 0 | 0 |
| Alcoholic drink | 0 | 0 | 0 | 0 | 10 | 7.8 | 0 | 0 | 0 | 0 | 0 | 0 |
| Gambling | 0 | 0 | 5 | 0.6 | 0 | 0 | 0 | 0 | 3 | 1.9 | 18 | 1.1 |
| Other | 70 | 61.4 | 623 | 72.5 | 56 | 43.8 | 981 | 75.8 | 101 | 63.1 | 1,348 | 82.4 |
| Empty panel | 22 | 19.3 | 23 | 2.7 | 16 | 12.5 | 85 | 6.6 | 5 | 3.1 | 120 | 7.3 |
| *Food group^a^* | | | | | | | | | | | | |
| Low(non) caloric drinks | 2 | 9.1 | 9 | 4.3 | 14 | 25 | 19 | 8.4 | 5 | 9.8 | 12 | 8.1 |
| Biscuits/cakes | 0 | 0 | 20 | 9.6 | 1 | 1.8 | 5 | 2.2 | 2 | 3.9 | 0 | 0 |
| Dairy and alternatives | 0 | 0 | 15 | 7.2 | 0 | 0 | 41 | 17.7 | 9 | 17.7 | 17 | 11.4 |
| Grains, potatoes, fruit & veg | 3 | 13.6 | 17 | 8.2 | 5 | 8.9 | 15 | 6.6 | 0 | 0 | 23 | 15.3 |
| Chocolate and sweets | 1 | 4.5 | 27 | 13 | 1 | 1.8 | 2 | 0.9 | 5 | 9.8 | 2 | 0.01 |
| Crisps/savoury snacks | 0 | 0 | 1 | 0.5 | 1 | 1.8 | 1 | 0.4 | 4 | 7.8 | 0 | 0 |
| Fast food | 12 | 54.6 | 115 | 55.3 | 12 | 21.4 | 31 | 13.3 | 14 | 27.4 | 8 | 5.3 |
| Fruit juice | 2 | 9.1 | 2 | 1 | 1 | 1.8 | 30 | 13.1 | 0 | 0 | 0 | 0 |
| Sugary drinks | 2 | 9.1 | 2 | 1 | 3 | 5.4 | 38 | 16.4 | 5 | 9.8 | 18 | 12.1 |
| Desserts | 0 | 0 | 0 | 0 | 8 | 14.3 | 46 | 20.4 | 3 | 5.9 | 6 | 4 |
| Alcoholic drinks | 0 | 0 | 0 | 0 | 10 | 17.9 | 0 | 0 | 0 | 0 | 0 | 0 |
| Tea/coffee/water | 0 | 0 | 0 | 0 | 0 | 0 | 0 | 0 | 4 | 7.8 | 59 | 36.6 |
| Processed meat, poultry, fish | 0 | 0 | 0 | 0 | 0 | 0 | 0 | 0 | 0 | 0 | 5 | 3.3 |

*^a^ Percentage is out of total food, non-alcoholic and food and non-alcoholic drink adverts in each area at each time period.*

**Supplementary Table 2**: Description of total adverts and adverts for unhealthy commodities at T0 and T1 in Bristol and SG, using data from GSV.

|  | T0(n=920) | | | | T1 (n=1,110) | | | |
| --- | --- | --- | --- | --- | --- | --- | --- | --- |
|  | **02/2022 - 07/2022** | | | | **04/2023 - 07/2023** | | | |
|  | **SG** | | **Bristol** | | **SG** | | **Bristol** | |
|  | **N** | **%** | **N** | **%** | **N** | **%** | **N** | **%** |
| Number of Advert Panels | 44 | 100 | 876 | 100 | 124 | 100 | 986 | 100 |
| Adverts for Unhealthy commodities |  |  |  |  |  |  |  |  |
| Yes | 4 | 9.1 | 77 | 8.8 | 17 | 13.7 | 43 | 4.4 |
| No | 30 | 68.2 | 698 | 79.7 | 85 | 68.6 | 642 | 65.1 |
| Empty panel | 2 | 4.6 | 33 | 3.8 | 1 | 0.8 | 72 | 7.3 |
| Missing | 8 | 18.2 | 68 | 7.8 | 21 | 16.9 | 229 | 23.2 |

**Supplementary Table 3:** Description of adverts for unhealthy commodities in Bristol and SG, at T0, T1 and T2.

|  | T0 (n=973) 02/2022 - 07/2022 | | | | T1 (n=1,423) 04/2023 - 07/2023 | | | | T2 (n=1,796) 11/2023 - 02/2024 | | | |
| --- | --- | --- | --- | --- | --- | --- | --- | --- | --- | --- | --- | --- |
|  | **SG (N=114)** | | **Bristol (N=859)** | | **SG (N=128)** | | **Bristol (N=1,295)** | | **SG (N=160)** | | **Bristol (N=1,636)** | |
|  | **N** | **%** | **N** | **%** | **N** | **%** | **N** | **%** | **N** | **%** | **N** | **%** |
| Adverts for unhealthy commodities^a^ | 1 | 0.9 | 97 | 11.8 | 26 | 20.3 | 46 | 3.6 | 29 | 18.1 | 13 | 0.8 |
| Type of Advert^b^ | | | | | | | | | | | | |
| Food | 1 | 100 | 94 | 96.9 | 8 | 30.8 | 15 | 32.6 | 21 | 65.6 | 13 | 100 |
| Non-alcoholic drink | 0 | 0.0 | 2 | 2.1 | 6 | 23.1 | 30 | 65.2 | 8 | 25.0 | 0 | 0.0 |
| Food and non-alcoholic drink | 0 | 0.0 | 0 | 0.0 | 2 | 7.7 | 1 | 2.2 | 0 | 0.0 | 0 | 0.0 |
| Alcoholic drink | 0 | 0.0 | 0 | 0.0 | 10 | 38.5 | 0 | 0.0 | 0 | 0.0 | 0 | 0.0 |
| Gambling | 0 | 0.0 | 1 | 1.0 | 0 | 0.0 | 0 | 0.0 | 0 | 0.0 | 0 | 0.0 |
| Food group^c^ | | | | | | | | | | | | |
| Low(non) caloric drinks | 0 | 0.0 | 0 | 0.0 | 0 | 0.0 | 0 | 0.0 | 0 | 0.0 | 0 | 0.0 |
| Biscuits/cakes | 0 | 0.0 | 20 | 20.8 | 1 | 3.8 | 0 | 0.0 | 2 | 6.9 | 0 | 0.0 |
| Dairy and alternatives | 0 | 0.0 | 6 | 6.3 | 0 | 0.0 | 0 | 0.0 | 7 | 24.1 | 7 | 53.9 |
| Grains, potatoes, fruit & vegetables | 0 | 0.0 | 0 | 0.0 | 0 | 0.0 | 2 | 2.2 | 0 | 0.0 | 0 | 0.0 |
| Chocolate and sweets | 1 | 100 | 23 | 24.0 | 2 | 7.7 | 0 | 0.0 | 5 | 17.2 | 1 | 7.7 |
| Crisps/savoury snacks | 0 | 0.0 | 1 | 1.0 | 1 | 3.9 | 2 | 2.2 | 5 | 17.2 | 0 | 0.0 |
| Fast food | 0 | 0.0 | 45 | 46.9 | 4 | 15.4 | 11 | 23.9 | 8 | 27.6 | 4 | 30.8 |
| Fruit juice | 0 | 0.0 | 0 | 0.0 | 0 | 0.0 | 0 | 0.0 | 0 | 0.0 | 0 | 0.0 |
| Sugary drinks | 0 | 0.0 | 1 | 1.0 | 4 | 15.4 | 29 | 63.0 | 0 | 0.0 | 0 | 0.0 |
| Desserts | 0 | 0.0 | 0 | 0.0 | 4 | 15.4 | 3 | 6.5 | 2 | 6.9 | 0 | 0.0 |
| Alcoholic drinks | 0 | 0.0 | 0 | 0.0 | 10 | 38.5 | 0 | 0.0 | 0 | 0.0 | 1 | 7.7 |
| Tea/coffee/water | 0 | 0.0 | 0 | 0.0 | 0 | 0.0 | 0 | 0.0 | 0 | 0.0 | 0 | 0.0 |
| Processed meat, poultry, fish | 0 | 0.0 | 0 | 0.0 | 0 | 0.0 | 0 | 0.0 | 0 | 0.0 | 0 | 0.0 |

*^a^ Percentage is out of total adverts in each area at each time period.*

*^b^Percentage is out of total adverts that are subject to restrictions in each area at each time period.*

*^c^Percentage is out of total food, non-alcoholic and food and non-alcoholic drink adverts in each area at each time period.*

*N (%) missing due to missing information on serving/grams of product and subsequently NPM could not be calculated.*

*T0: Bristol – 59 (7%), SG – 12 (11%)*

*T1: Bristol – 9 (0.7%), SG – 5 (4%)*

*T2: Bristol – 6 (0.4%), SG– 3 (2%)*

Supplementary Table 4: The top 5 advertised organisations/brands at T0, T1 and T2 in a) Bristol and b) South Gloucestershire.

a) **Bristol**

| T0 (02/2022 - 07/2022) | | | | | | | |
| --- | --- | --- | --- | --- | --- | --- | --- |
| Other adverts (N=648) | | | | **Adverts for unhealthy commodities (N=96)** | | | |
| Types of advertised brands | **Example brands** | **N** | **%** | **Types of advertised brands** | **Example brands** | **N** | **%** |
| Government/Council | Covid related content | 132 | 19.5 | Fast food chains | McDonald's, Burger King, KFC | 45 | 44.6 |
| Careers/Business/Pension | Pensionbee, Reed, CV Library | 104 | 15.4 | Restaurants/Cafes/Delivery | Costa Coffee | 5 | 5 |
| Charities | MacMillan, Dementia Connect | 99 | 14.6 | Food/drink products | LU, Cadbury, Coca Cola | 45 | 44.6 |
| Transport/Travel | Metrobus, TravelWest | 54 | 8 |  |  |  |  |
| Supermarkets | Tesco, CoOp | 34 | 5 |  |  |  |  |
| T1 (04/2023 - 07/2023) | | | | | | | |
| Other adverts (N=1,138) | | | | **Adverts for unhealthy commodities (N=46)** | | | |
| Types of advertised brands | **Example brands** | **N** | **%** | **Types of advertised brands** | **Example brands** | **N** | **%** |
| Transport/Travel | Travel West, First Bus, Stagecoach Bus | 433 | 37.5 | Food/drink products | Coca Cola, San Pellegrino | 33 | 71.7 |
| Food/drink products | Muller, Cawston Press, Oatly, Coca Cola | 169 | 14.6 | Fast food chains | McDonald's, Subway | 7 | 15.2 |
| Media/TV/Entertainment | Google, Channel 5, Disney | 88 | 7.6 | Restaurants/Cafes/Delivery | Uber Eats | 5 | 10.9 |
| Shops (non-food) | B&Q, boohooMAN | 70 | 6 | Supermarkets | Tesco | 1 | 2.2 |
| Mobiles/Mobile networks | Samsung, Vodafone | 66 | 5.7 |  |  |  |  |
| T2 (11/2023 - 02/2024) | | | | | | | |
| Other adverts (N=1,497) | | | | **Adverts for unhealthy commodities (N=13)** | | | |
| Types of advertised brands | **Example brands** | **N** | **%** | **Types of advertised brands** | **Example brands** | **N** | **%** |
| Transport/Travel | TravelWest, Metrobus | 400 | 26.7 | Food/drink products | Oxo, Muller, KitKat | 8 | 61.5 |
| Government/Council | West of England Combined Authority | 193 | 12.9 | Fast food chains | Subway, McDonald's | 4 | 30.8 |
| Charities | Teenage Cancer Trust, Gamble Aware | 144 | 9.6 | Supermarkets | Aldi | 1 | 7.7 |
| Shops (non-food) | Amazon, Very, Boohooman | 140 | 9.3 |  |  |  |  |
| Food/drink products | PG Tips, Pepsi, Coca Cola, Heinz | 121 | 8.0 |  |  |  |  |

b) **South Gloucestershire**

| T0 (02/2022 - 07/2022) | | | | | | | |
| --- | --- | --- | --- | --- | --- | --- | --- |
| Other adverts (N=66) | | | | **Adverts for unhealthy commodities (N=1)** | | | |
| Types of advertised brands | **Example brands** | **N** | **%** | **Types of advertised brands** | **Example brands** | **N** | **%** |
| Household products/services | Evri | 16 | 20.3 | Supermarkets | Tesco | 1 | 100 |
| Charities | Prostate Cancer UK, Teenage Cancer Trust | 13 | 16.5 |  |  |  |  |
| Banks/Investment | Lloyds | 7 | 8.9 |  |  |  |  |
| Food/drink products | Coca Cola, Robinson's | 7 | 8.9 |  |  |  |  |
| Advert companies | Clear Channel | 4 | 5 |  |  |  |  |
| T1 (04/2023 - 07/2023) | | | | | | | |
| Other adverts (N=81) | | | | **Adverts for unhealthy commodities (N=26)** | | | |
| Types of advertised brands | **Example brands** | **N** | **%** | **Types of advertised brands** | **Example brands** | **N** | **%** |
| Food/drink products | Lucozade, Dolmio, Muller | 24 | 29.6 | Food/drink products | Coca Cola, Pepsi, Ferrero Rocher | 11 | 42.3 |
| Media/TV/Entertainment | Channel 5, Google | 16 | 19.8 | Alcohol | Strongbow, Thatcher's | 10 | 38.5 |
| Transport/Travel | TravelWest | 12 | 14.8 | Fast Food chains | McDonald's, Subway | 5 | 19.2 |
| Mobiles/Mobile networks | Vodafone, Smarty | 8 | 9.9 |  |  |  |  |
| Banks/Investment | Lloyds, Nationwide | 5 | 6.2 |  |  |  |  |
| T2 (11/2023 - 02/2024) | | | | | | | |
| Other adverts (N=120) | | | | **Adverts for unhealthy commodities (N=29)** | | | |
| Types of advertised brands | **Example brands** | **N** | **%** | **Types of advertised brands** | **Example brands** | **N** | **%** |
| Transport/Travel | TravelWest, Metrobus | 46 | 38.3 | Fast Food chains | McDonald's | 11 | 34.4 |
| Food/drink products | Coca Cola, PG Tips, Pepsi | 16 | 13.3 | Food/drink products | Nestle, McCoy's | 10 | 31.2 |
| Media/TV/Entertainment | Playstation | 10 | 8.3 | Restaurants/Cafes/Delivery | Costa Coffee | 5 | 15.6 |
| Charities | MacMillan, The Prince's Trust | 9 | 7.5 | Supermarkets | Tesco | 3 | 9.4 |
| Government/Council | West of England Combined Authority | 9 | 7.5 |  |  |  |  |

**Supplementary Table 5**: Description of adverts subject to restrictions a) close and b) not close to the Bristol-SG border (within 400m).

|  | a) Adverts for unhealthy commodities (close to the borders) | | | | | |  | b) Adverts for unhealthy commodities (not close to the borders) | | | | | |
| --- | --- | --- | --- | --- | --- | --- | --- | --- | --- | --- | --- | --- | --- |
| T0 | **S Glous (N=0)** | | | **Bristol (N=5)** | | |  | **S Glous (N=1)** | | | **Bristol (N=92)** | | |
|  | **Brand** | **N** | **%** | **Brand** | **N** | **%** |  | **Brand** | **N** | **%** | **Brand** | **N** | **%** |
|  |  | | | Food/drink products | 4 | 80 |  | Supermarket | 1 | 100 | Fast food chains | 44 | 47.8 |
|  |  |  |  | Fast food chains | 1 | 20 |  |  | | | Food/drink products | 42 | 45.7 |
|  |  |  |  |  | | |  |  |  |  | Restaurants/cafes | 5 | 5.4 |
|  |  |  |  |  |  |  |  |  |  |  | Gambling | 1 | 1.1 |
| T1 | **S Glous (N=4)** | | | **Bristol (N=3)** | | |  | **S Glous (N=22)** | | | **Bristol (n=43)** | | |
|  | **Brand** | **N** | **%** | **Brand** | **N** | **%** |  | **Brand** | **N** | **%** | **Brand** | **N** | **%** |
|  | Food/drink products | 3 | 75 | Food/drink products | 3 | 100 |  | Food/drink products | 12 | 54.6 | Food/drink products | 30 | 69.8 |
|  | Fast food chains | 1 | 25 |  | | |  | Alcohol | 6 | 27.3 | Fast food chains | 7 | 16.3 |
|  |  | | |  |  |  |  | Fast food chains | 4 | 18.2 | Restaurants/cafes | 5 | 11.6 |
|  |  |  |  |  |  |  |  |  | | | Supermarket | 1 | 2.3 |
| T2 | **S Glous (N=3)** | | | **Bristol (N=0)** | | |  | **S Glous (N=26)** | | | **Bristol (N=13)** | | |
|  | **Brand** | **N** | **%** | **Brand** | **N** | **%** |  | **Brand** | **N** | **%** | **Brand** | **N** | **%** |
|  | Fast food chains | 1 | 33.3 |  | | |  | Fast food chains | 10 | 38.5 | Food/drink products | 8 | 61.5 |
|  | Food/drink products | 1 | 33.3 |  |  |  |  | Food/drink products | 9 | 34.6 | Fast food chains | 4 | 30.8 |
|  | Restaurants/cafes | 1 | 33.3 |  |  |  |  | Restaurants/cafes | 4 | 15.4 | Supermarket | 1 | 7.7 |
|  |  | | |  |  |  |  | Supermarket | 3 | 11.5 |  | | |

Supplementary Table 6: Proximity of adverts (within 100m, 200m and 400m) to Bristol and South Gloucestershire schools (both primary and secondary) at T0, T1 and T2

|  | T0 (n=973) 02/2022 - 07/2022 | | | | T1 (n=1,423) 04/2023 - 07/2023 | | | | T2 (n=1,796) 11/2023 - 02/2024 | | | |
| --- | --- | --- | --- | --- | --- | --- | --- | --- | --- | --- | --- | --- |
|  | **SG (N=114)** | | **Bristol (N=859)** | | **SG (N=128)** | | **Bristol (N=1,295)** | | **SG (N=160)** | | **Bristol (N=1,636)** | |
|  | **N** | **%** | **N** | **%** | **N** | **%** | **N** | **%** | **N** | **%** | **N** | **%** |
| *100m* | | | | | | | | | | | | |
| Total adverts ^a^ | 8 | 7.0 | 27 | 3.1 | 10 | 7.8 | 32 | 2.5 | 12 | 7.5 | 46 | 2.8 |
| Food/drink advert ^b^ | 1 | 12.5 | 2 | 7.4 | 3 | 30.0 | 1 | 3.1 | 4 | 33.3 | 2 | 4.4 |
| Unhealthy commodity advert ^b^ | 0 | 0.0 | 0 | 0.0 | 1 | 10.0 | 0 | 0.0 | 0 | 0.0 | 1 | 2.2 |
| Food/drink advert (HFSS) ^b^ | 0 | 0.0 | 0 | 0.0 | 1 | 10.0 | 0 | 0.0 | 0 | 0.0 | 1 | 2.2 |
| Alcohol advert ^b^ | 0 | 0.0 | 0 | 0.0 | 0 | 0.0 | 0 | 0.0 | 0 | 0.0 | 0 | 0.0 |
| Gambling advert ^b^ | 0 | 0.0 | 0 | 0.0 | 0 | 0.0 | 0 | 0.0 | 0 | 0.0 | 0 | 0.0 |
| *200m* | | | | | | | | | | | | |
| Total adverts ^a^ | 24 | 21.1 | 81 | 9.4 | 34 | 26.6 | 140 | 10.8 | 45 | 28.1 | 166 | 10.1 |
| Food/drink advert ^b^ | 1 | 4.2 | 6 | 7.4 | 8 | 23.5 | 23 | 16.4 | 8 | 17.8 | 16 | 9.6 |
| Unhealthy commodity advert ^b^ | 0 | 0.0 | 0 | 0.0 | 1 | 2.9 | 6 | 4.3 | 4 | 8.9 | 4 | 2.4 |
| Food/drink advert (HFSS) ^b^ | 0 | 0.0 | 0 | 0.0 | 1 | 2.9 | 6 | 4.3 | 4 | 8.9 | 4 | 2.4 |
| Alcohol advert ^b^ | 0 | 0.0 | 0 | 0.0 | 0 | 0.0 | 0 | 0.0 | 0 | 0.0 | 0 | 0.0 |
| Gambling advert ^b^ | 0 | 0.0 | 0 | 0.0 | 0 | 0.0 | 0 | 0.0 | 2 | 4.4 | 5 | 3.0 |
| *400m* | | | | | | | | | | | | |
| Total adverts ^a^ | 64 | 56.1 | 438 | 51.0 | 74 | 57.8 | 757 | 558.5 | 93 | 58.1 | 974 | 59.5 |
| Food/drink advert ^b^ | 8 | 12.5 | 73 | 16.7 | 20 | 27.0 | 122 | 16.1 | 23 | 24.7 | 88 | 9.0 |
| Unhealthy commodity advert^b^ | 1 | 1.6 | 26 | 5.9 | 11 | 14.9 | 24 | 3.2 | 13 | 14.0 | 8 | 0.8 |
| Food/drink advert (HFSS) ^b^ | 1 | 1.6 | 26 | 5.9 | 8 | 10.8 | 24 | 3.2 | 13 | 14.0 | 8 | 0.8 |
| Alcohol advert ^b^ | 0 | 0.0 | 0 | 0.0 | 3 | 4.1 | 0 | 0.0 | 0 | 0.0 | 0 | 0.0 |
| Gambling advert ^b^ | 0 | 0.0 | 1 | 0. 2 | 0 | 0.0 | 0 | 0.0 | 2 | 2.2 | 0 | 0.0 |

*^a^ Percentage is out of the total adverts located in each area at each time period*

*^b^ Percentage is out of the total adverts located in each buffer zone, in each area at each timepoint*

Supplementary Figure 2: Adverts for unhealthy commodities appealing to children or adolescents in a) Bristol and b) SG.

a) Bristol

|  | Appealing to children or adolescents | Not appealing to children or adolescents |
| --- | --- | --- |
| T0 Unhealthy commodity adverts | 2.97 | 97 |
| T0 Other adverts | 0.9 | 99.1 |
| T1 Unhealthy commodity adverts | 21.7 | 78.3 |
| T1 Other adverts | 5.7 | 94.1 |
| T2 Unhealthy commodity adverts | 0 | 100 |
| T2 Other adverts | 4.2 | 95.8 |

b) South Gloucestershire

|  | Appealing to children or adolescents | Not appealing to children or adolescents |
| --- | --- | --- |
| T0 Unhealthy commodity adverts | 0 | 100 |
| T0 Other adverts | 3.8 | 96.2 |
| T1 Unhealthy commodity adverts | 7.7 | 92.3 |
| T1 Other adverts | 9.9 | 90.1 |
| T2 Unhealthy commodity adverts | 18.8 | 81.3 |
| T2 Other adverts | 6.7 | 93.3 |
